# Drug sensitivity profiling identifies potential therapies for lymphoproliferative disorders with overactive JAK/STAT3 signaling

**DOI:** 10.18632/oncotarget.22178

**Published:** 2017-10-31

**Authors:** Heikki Kuusanmäki, Olli Dufva, Elina Parri, Arjan J. van Adrichem, Hanna Rajala, Muntasir M. Majumder, Bhagwan Yadav, Alun Parsons, Wing C. Chan, Krister Wennerberg, Satu Mustjoki, Caroline A. Heckman

**Affiliations:** ^1^ Institute for Molecular Medicine Finland, Helsinki Institute of Life Science, University of Helsinki, Helsinki, Finland; ^2^ Hematology Research Unit, Helsinki University Hospital Comprehensive Cancer Center, Helsinki, Finland; ^3^ Department of Pathology, City of Hope National Medical Center, Duarte, CA, USA; ^4^ Department of Clinical Chemistry and Hematology, University of Helsinki, Helsinki, Finland

**Keywords:** STAT3 mutation, Hsp90 and JAK inhibitors, high-throughput compound screening, hematological malignancy

## Abstract

Constitutive JAK/STAT3 signaling contributes to disease progression in many lymphoproliferative disorders. Recent genetic analyses have revealed gain-of-function *STAT3* mutations in lymphoid cancers leading to hyperactivation of STAT3, which may represent a potential therapeutic target. Using a functional reporter assay, we screened 306 compounds with selective activity against various target molecules to identify drugs capable of inhibiting the cellular activity of STAT3. Top hits were further validated with additional models including STAT3-mutated natural killer (NK)-cell leukemia/lymphoma cell lines and primary large granular lymphocytic (LGL) leukemia cells to assess their ability to inhibit STAT3 phosphorylation and STAT3 dependent cell viability. We identified JAK, mTOR, Hsp90 and CDK inhibitors as potent inhibitors of both WT and mutant STAT3 activity. The Hsp90 inhibitor luminespib was highly effective at reducing the viability of mutant STAT3 NK cell lines and LGL leukemia patient samples. Luminespib decreased the phosphorylation of mutant STAT3 at Y705, whereas JAK1/JAK2 inhibitor ruxolitinib had reduced efficacy on mutant STAT3 phosphorylation. Additionally, combinations involving Hsp90, JAK and mTOR inhibitors were more effective at reducing cell viability than single agents. Our findings show alternative approaches to inhibit STAT3 activity and suggest Hsp90 as a therapeutic target in lymphoproliferative disorders with constitutively active STAT3.

## INTRODUCTION

STAT3 is a transcription factor that participates in tumorigenesis by upregulating expression of cancer promoting genes such as anti-apoptotic BCL2-family members (Mcl-1, Bcl-2, Bcl-X_L_) and cell cycle regulators (c-Myc, Cyclin D1) [1, 2]. In lymphoproliferative malignancies, JAK/STAT3 signaling is commonly deregulated and thus an attractive target for treatment [3-6]. Mechanisms leading to overactive STAT3 signaling include fusion genes with kinase activity, loss of phosphatases, chronic stimulation of cytokine receptors via extrinsic cytokines, and activating mutations in upstream tyrosine kinases [7, 8]. In addition, recent genomic profiling studies have identified *STAT3* mutations in a substantial fraction of lymphoid malignancies, including large granular lymphocytic (LGL) leukemia (prevalence 40%), CD30+ diffuse large B-cell lymphoma (6%), T-cell lymphomas (7%), multiple myeloma (4%), anaplastic large cell lymphoma (10%), natural killer (NK) cell lymphoma (6%) and intestinal T-cell lymphomas (12%) [8-18]. The majority are gain-of-function mutations, such as Y640F and D661V, and occur in the SH2 domain of the STAT3 protein leading to increased tyrosine 705 phosphorylation (Y705), which is needed for protein dimerization and activation [19].

Current approaches to inhibit wild-type (WT) STAT3 activation include JAK inhibitors such as ruxolitinib and tofacitinib and direct blocking of STAT3 dimerization with SH2 domain antagonists such as Stattic, LLL12, OPB-51602 and OPB-31121 [20-24]. However, selective STAT3 SH2 domain antagonists have not yet yielded useful therapies partly because STATs are pharmacologically challenging targets. Other recent studies involving high-throughput compound screens have identified piperlongumine and methotrexate as potential JAK/STAT3 pathway inhibitors [25, 26]. However, earlier studies have not systematically examined whether targeted compounds, including JAK inhibitors and STAT3 antagonists, are effective at reducing mutant STAT3 activity. Furthermore, it is not known whether mutant STAT3 confers a distinct drug response profile compared to WT STAT3.

To identify targeted drugs that can potentially inhibit constitutively active STAT3 signaling, we assessed the activity of 306 approved and investigational agents in a STAT3 luciferase reporter assay. Positive hits were further validated in different models including STAT3 mutation-containing Ba/F3 cells, NK cell leukemia/lymphoma cells and LGL leukemia patient samples. Besides blocking JAK activity, our results indicate that inhibition of other molecules, such as Hsp90, may have greater impact on mutant STAT3, and could be investigated as therapeutic options for lymphoproliferative diseases with STAT3 mutations.

## RESULTS

### mTOR, JAK, Hsp90 and CDK inhibitors decrease cellular activity of mutant STAT3

We prescreened 306 compounds with selective activity against various target molecules (Supplementary Table 1) to identify direct or indirect inhibitors of STAT3 activity and to determine whether activating STAT3 mutations confer a drug response profile distinct from WT STAT3. For this screen we used HEK293 cells containing a luciferase reporter under the control of a STAT3 inducible element (HEK293-SIE cells) stably expressing either WT STAT3 or the most common and hyperactive mutant form of STAT3 (Y640F) [9]. In the absence of interleukin stimulation, luciferase activity was high in mutant STAT3 containing cells and was further augmented in the presence of IL6. In contrast, IL6 was required to induce luciferase activity in WT STAT3 containing cells (Supplementary Figure 1).

**Figure 1:**
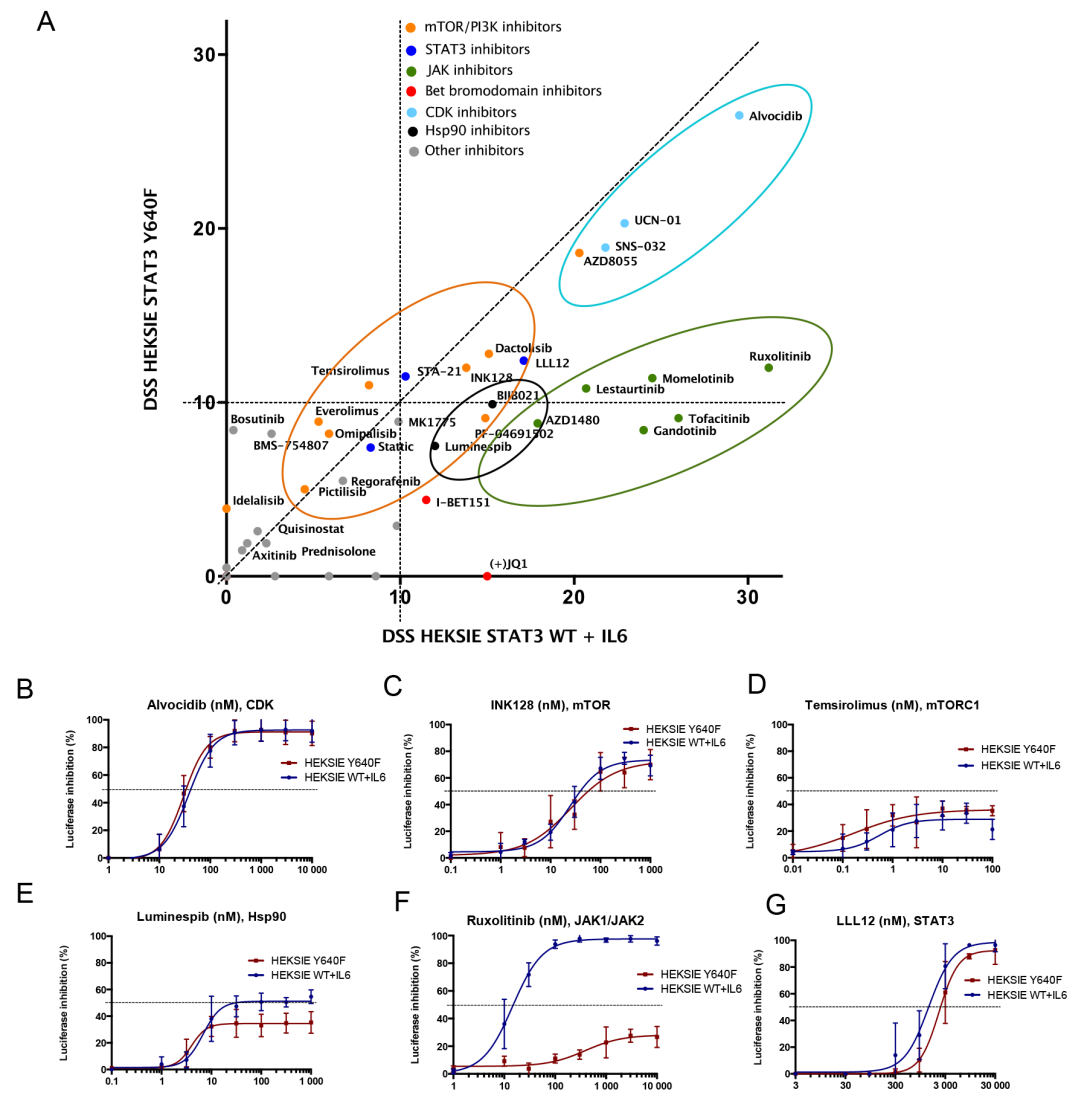
mTOR, CDK, Hsp90 and JAK inhibitors reduce STAT3-induced reporter activity **(A)** Correlation of drug sensitivity scores (DSS) indicating luciferase activity inhibition in HEK293-SIE cells after 6 h compound treatment. DSS is a quantitative measurement of a drug response based on the area under the curve (AUC) with further normalization. Y-axis: HEK293-SIE cells stably expressing constitutively active Y640F STAT3. X-axis: HEK293-SIE stably expressing WT STAT3 + IL6 stimulation (100 ng/ml) 15 min before addition of the compounds. The experiment was repeated two times and mean DSS values are shown in the plot. **(B-G)** Dose response curves showing inhibition of luciferase activity after treatment with alvocidib (CDK inhibitor), INK128 (mTORC1/mTORC2 inhibitor), temsirolimus (mTORC1 inhibitor), luminespib (Hsp90 inhibitor), ruxolitinib (JAK1/2 inhibitor) and LLL12 (STAT3 inhibitor). Error bars represent ±SD, n=3.

Results from the initial screen indicated efficacy of several agents against both WT and mutant STAT3 activity (data not shown). Based on these results we designed a smaller panel of 62 agents containing targeted compounds that effectively reduced STAT3 activity, including cyclin-dependent kinase (CDK), mammalian target of rapamycin (mTOR), heat shock protein 90 (Hsp90), and Janus kinase (JAK) inhibitors (Figure 1A, Supplementary Table 1). Dose response curves and half maximal inhibitory concentration (IC50) values of the 62 compounds studied in more detail are presented in Supplementary Table 2. CDK, mTOR and Hsp90 inhibitors showed similar activity between mutant and WT STAT3 whereas JAK inhibitors had clearly reduced efficacy against mutant STAT3 (Figure 1A-1F). Interestingly, the Src-family kinase inhibitor bosutinib and the insulin-like growth factor 1 receptor inhibitor BMS-754807 inhibited only mutant STAT3, whereas the BET bromodomain inhibitor JQ-1 was only effective against WT STAT3 induced through the IL6 receptor, demonstrating that STAT3 mutation can alter sensitivity to certain compounds (Figure 1A, Supplementary Table 2). The small molecule STAT3 inhibitors (STA-21, LLL12, Stattic), which are SH2 domain antagonists, reduced both WT and mutant STAT3 activity indicating that the mutation does not alter the binding capabilities of these drugs (Figure 1G, Supplementary Table 2). All together, these observations suggest that in the absence of extrinsic cytokine stimulation JAK inhibition alone is not sufficient to block the cellular activity of mutant STAT3, however, the activity can be reduced by other targeted compounds.

### Hsp90 inhibition abrogates mutant STAT3 phosphorylation

**Figure 2:**
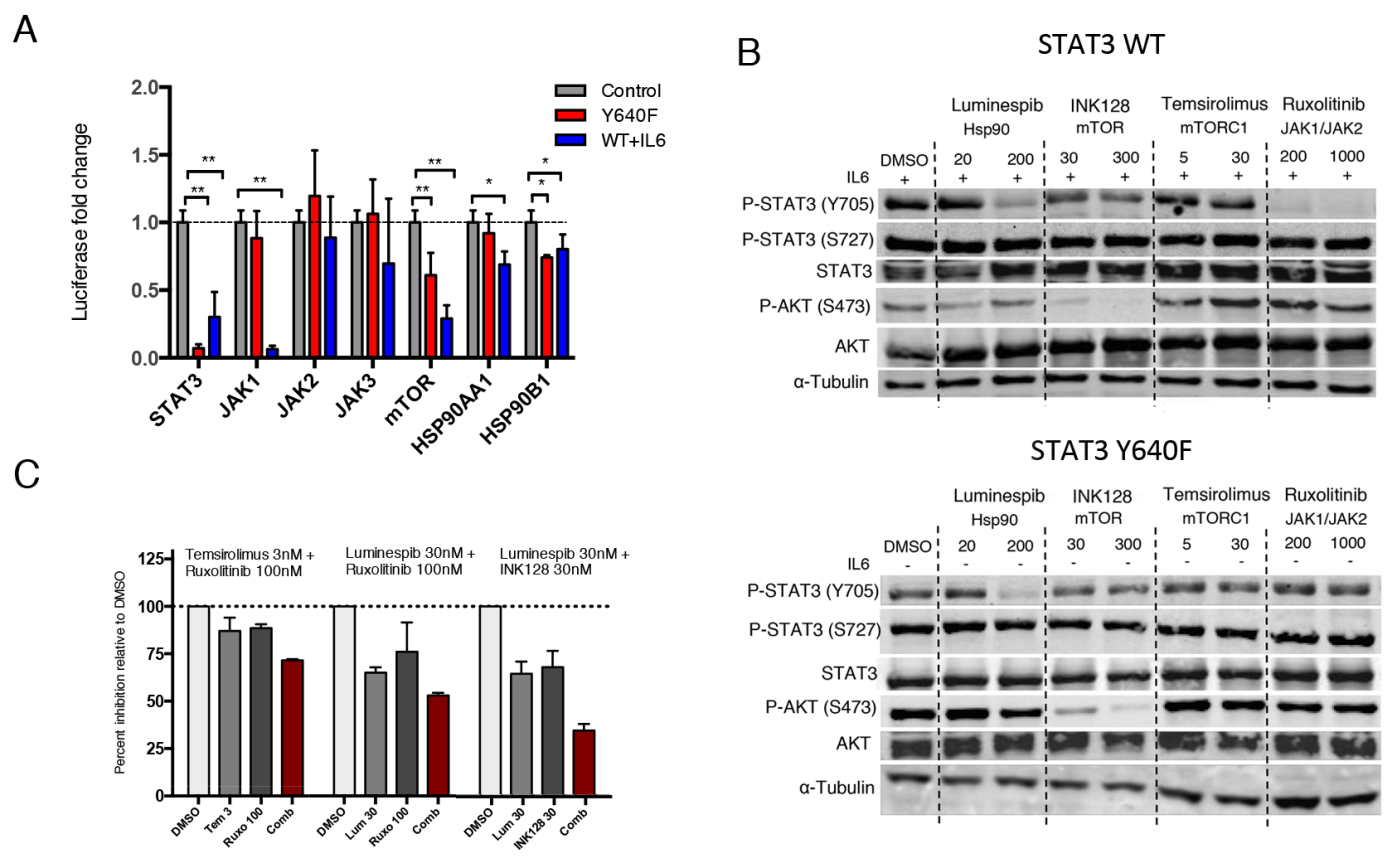
Hsp90 inhibition abrogates both mutant and WT STAT3 activity and phosphorylation **(A)** Silencing of target proteins using three different siRNAs for each gene. Luciferase activity was measured after 3 days and are relative to mock siRNA treated cells. Both cell lines were independently normalized to their corresponding mock treated cells. The mean values ± S.D. of 2 independent experiments are presented in the graph, ^*^P<0.05, ^**^P<0.01 (Mann-Whitney U-test). **(B)** WT or mutant STAT3 expressing HEK293-SIE cells were treated with the indicated compounds or DMSO for 8 h, followed by western blotting for Y705 P-STAT3, S727 P-STAT3, STAT3, S473 P-AKT, AKT and α-Tubulin. WT STAT3 expressing cells were stimulated with 100ng/ml IL-6 before treatment. **(C)** Effect of three different drug combinations on mutant STAT3-dependent reporter signal. Compounds were incubated for 6 h followed by luciferase signal measurement and are relative to DMSO control. Histograms show the inhibitory effect of a drug combination and individual drugs of two independent experiments. Synergism scores and 2D contour plots from concentration matrix are presented in Supplementary Figure 2.

To confirm the involvement of the drug targets in mediating STAT3 activity, we investigated whether siRNA-mediated knockdown of JAK, Hsp90 and mTOR result in similar reduction of STAT3 activity compared to pharmacological inhibition. As a control, STAT3 knockdown caused over 80% reduction in luciferase activity in both cell line models. In line with the drug screening results, JAK1 knockdown caused over 90% inhibition in IL6-stimulated WT STAT3, whereas mutated STAT3 activity remained unaltered (Figure 2A). No clear effect was seen with JAK2 or JAK3 silencing, suggesting that IL6 stimulates STAT3 mainly through JAK1 in HEK293 cells. Consistent with the drug screening data, silencing of mTOR and HSP90 resulted in decreased activity of WT STAT3 as well as mutant STAT3 (Figure 2A). These results indicate that specific inhibition of the JAK kinases is not sufficient to block cellular activity of mutant STAT3. However, inhibition of mTOR or Hsp90 not only affects WT STAT3 activity, but can interfere with mutant STAT3 function as well.

To determine whether the observed drug effects are conveyed through decreased tyrosine or serine phosphorylation of STAT3, the cells were treated with ruxolitinib (JAK1/JAK2 inhibitor), luminespib (Hsp90 inhibitor), INK128 (mTORC1/mTORC2 inhibitor) and temsirolimus (mTORC1 inhibitor) for 8 h. Ruxolitinib effectively inhibited Y705 phosphorylation of WT STAT3 but not mutant STAT3 (Figure 2B). Notably, luminespib caused a clear decrease in Y705 phosphorylation of both mutant and WT STAT3 without affecting total STAT3, pAKT or S727 P-STAT3 levels (Figure 2B). In contrast, mTOR inhibitors were not able to reduce STAT3 phosphorylation, suggesting that their effect is mediated through a different mechanism. Results show that JAK kinases have only a small effect on transcriptional activity and phosphorylation of non-stimulated and overexpressed mutant STAT3, whereas Hsp90 is essential in the phosphorylation and activity of both WT and mutant STAT3.

Given that the compounds appeared to inhibit the transcriptional activity of mutant STAT3 by distinct mechanisms we next investigated whether drug combinations show synergistic effects. All of the tested combinations showed additive inhibition, but no striking synergism was observed (Supplementary Figure 2). The highest additive effect was seen when Hsp90 inhibitor luminespib was combined with mTOR inhibitor INK128 or JAK inhibitor ruxolitinib resulting in 40-65% inhibition of mutant STAT3 activity with low concentrations (Figure 2C). The additive inhibitory effects further suggest that the compounds inhibit STAT3 through non-redundant mechanisms.

### STAT3-dependent Ba/F3 cells are sensitive to mTOR and Hsp90 inhibition

**Figure 3:**
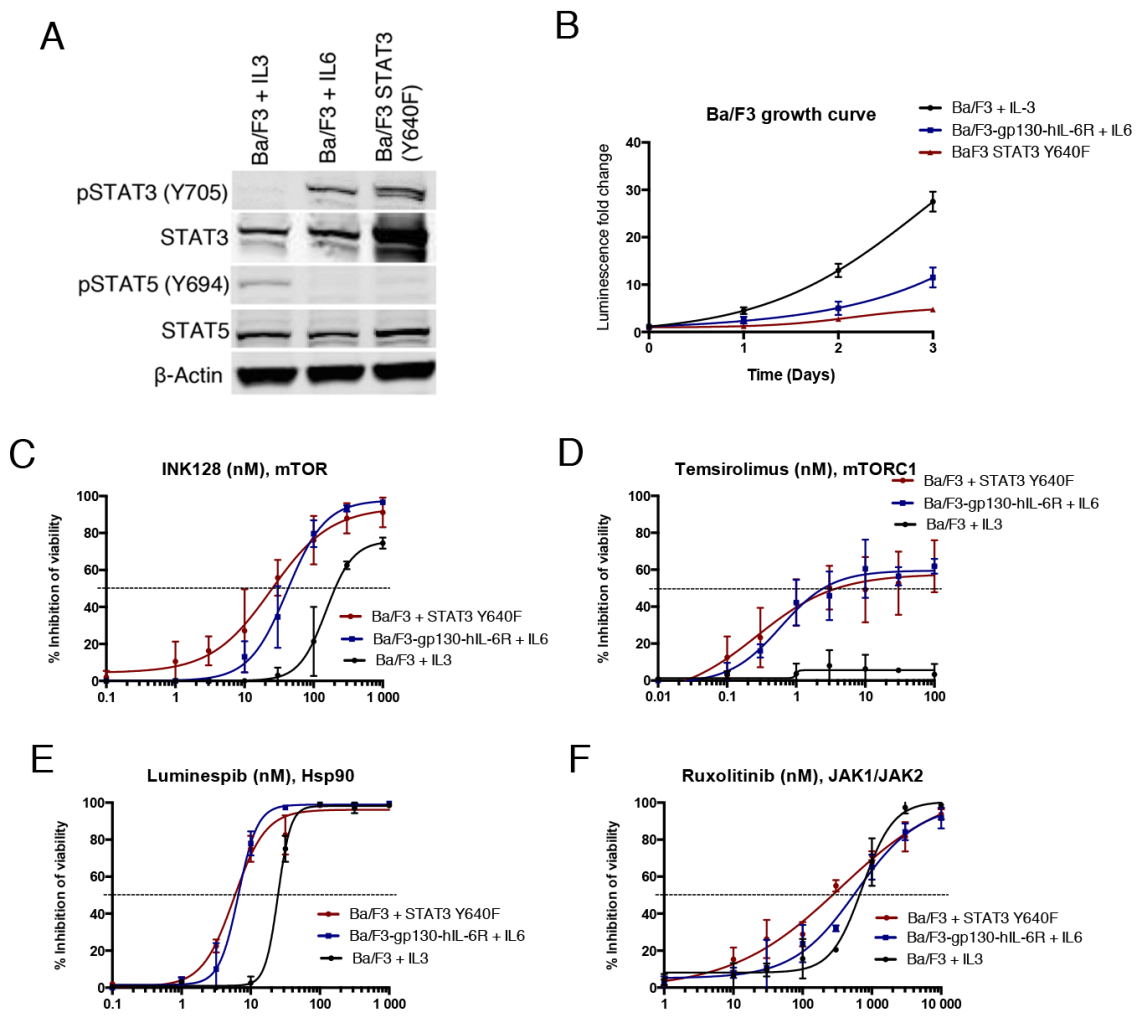
STAT3 dependent Ba/F3 cells show increased sensitivity to Hsp90 and mTOR inhibition **(A)** Western blot analysis showing Y705 P-STAT3 and total STAT3 protein levels of different Ba/F3 cell lines: Ba/F3 Y640F STAT3, Ba/F3 cells expressing gp130-hIL-6R cultured with IL6 (10 ng/ml), and parental Ba/F3 cells cultured with IL3 (10 ng/ml). **(B)** Growth curves of Ba/F3 cell lines. Cells were cultured for 3 days and viability was measured each day with the CTG assay. **(C-F)** Cell viability inhibition of Ba/F3 cells after treatment with INK128, temsirolimus, luminespib and ruxolitinib for 3 days. Cell viability was measured with CTG and error bars represent ±SD (n=3).

To determine if the observed drug effects on STAT3 function are associated with changes in STAT3-dependent cell growth and viability, we used the murine pro-B cell line Ba/F3. Ba/F3 cells are dependent on IL3 for survival, which activates signal transduction pathways mainly via JAK2 leading to STAT5B phosphorylation [27]. We transduced Ba/F3 cells with a construct expressing human Y640F STAT3, which was able to confer cytokine independency in contrast to WT STAT3. Furthermore, we used Ba/F3 cells which express human IL6R and gp130 subunits (Ba/F3-gp130-hIL-6R) to study IL6R/JAK/STAT3 associated proliferation [28]. Western blot analysis confirmed phosphorylation of STAT3 in both IL6-stimulated and mutant STAT3 expressing Ba/F3 cells, whereas STAT5 was phosphorylated in parental IL3-stimulated Ba/F3 cells (Figure 3A). Notably, the viability of IL3 and IL6 stimulated Ba/F3 cells increased over time to a greater extent when compared to mutant STAT3-transformed cells, indicating that mutant STAT3 alone is not a strong inducer of cell growth (Figure 3B).

In a 3-day cell viability assay, mutant STAT3 transformed and IL6-stimulated Ba/F3 cells were found to be sensitive to mTOR inhibition, in contrast to IL3-stimulated parental cells (Figure 3C and 3D). Furthermore, Hsp90 inhibitor luminespib was more effective in both STAT3-dependent models, although it also had substantial efficacy against parental Ba/F3 cells (Figure 3E). Although JAK inhibitors were not effective at blocking mutant STAT3 function in the luciferase reporter assays, ruxolitinib inhibited the viability of all three Ba/F3 cell models (Figure 3F). Taken together, these data demonstrate that STAT3-dependent Ba/F3 cells are highly sensitive to Hsp90 inhibition and show increased sensitivity to mTOR inhibition.

### NK cell lines harboring STAT3 mutation show increased mTOR but decreased JAK inhibitor sensitivity

**Figure 4:**
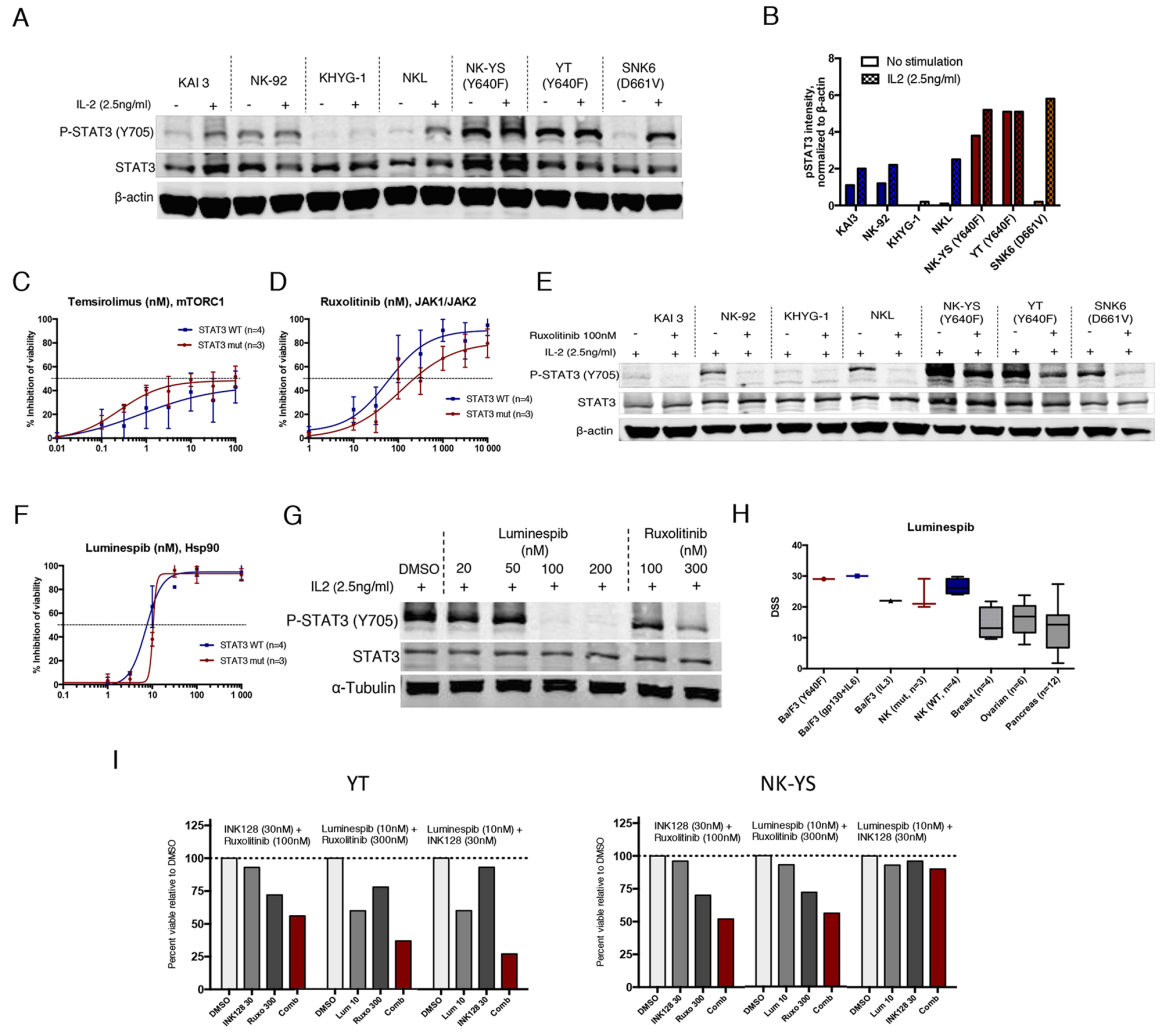
Mutant STAT3 NK cells show decreased sensitivity to JAK inhibitors but are highly sensitive to Hsp90 inhibition **(A)** Western blot analysis showing Y705 P-STAT3 and total STAT3 protein levels of 7 NK cell lines cultured in IL-2 (2.5ng/ml) or starved O/N without IL2. **(B)** Representative histogram of Y705 P-STAT3 band intensities analyzed from western blot images and quantified with the Odyssey imaging system software. Intensity values were normalized to β-actin **(C-D)** Inhibition of cell viability after treatment with temsirolimus and ruxolitinib. Mean of the inhibitory effect of three STAT3 mutated cell lines are presented in red line (YT, NK-YS, SNK6) and the blue line represents the mean of the WT STAT3 cell lines (KAI3, NK-92, KHYG-1, NKL). The experiment was repeated twice and error bars represent ±SD. **(E)** Western blot analysis showing Y705 P-STAT3 and total STAT3 protein levels of 7 NK cell lines cultured in IL-2 (2.5ng/ml). Half of the cells were treated with 100nM ruxolitinib for 6 h. **(F)** Inhibition of cell viability after treatment with luminespib. **(G)** Western blot analysis showing P-STAT3 levels of the NK-YS cell line after treatment with luminespib or ruxolitinib with increasing concentrations for 16 h and in the presence of IL2 (2.5ng/ml). **(H)** Mean and range of drug sensitivity scores (DSS) of cell lines originated from different tissues and treated with luminespib. All cell lines were incubated for 3 days and cell viability was measured with CTG assay. **(I)** Effect of 3 different drug combinations on the viability of NK-YS and YT cell lines during 3-day incubation. Histograms show the inhibitory effect of individual drugs and drug combinations from one chosen concentration. Synergism scores and 2D contour plots from concentration matrix are presented in Supplementary Figure 5.

Recent studies have shown that activating mutations to STAT3 frequently occur in NK/T-cell lymphomas, which are dependent on STAT3 activity for growth [17]. To validate our findings from cell lines engineered to overexpress STAT3, we tested 7 different NK cell leukemia/lymphoma cell lines with either WT STAT3 (KAI3, NK-92, KHYG-1 and NKL), or naturally occurring STAT3 mutations (YT and NK-YS, Y640F mutation; SNK6, D661V mutation) [29-31]. Most of the cell lines require IL2 (Supplementary Figure 3), which activates the JAK/STAT pathway, for proliferation and survival [32]. In the presence of IL2, STAT3 was highly phosphorylated in all mutant cell lines and remained phosphorylated upon overnight IL2 withdrawal in cells harboring Y640F mutation (Figure 4A and 4B).

The 7 cell lines were tested against the 62 compounds in a 3-day cell viability assay in the presence of IL2 with only JAK and mTORC1 inhibitors showing differences between mutant and WT STAT3 NK cells (Supplementary Figure 4). However, a statistically significant difference was not determined due to the limited number of cell lines. Nevertheless, as observed with Ba/F3 cells, mutant STAT3 NK cell lines were more sensitive to mTORC1 inhibition compared to WT STAT3 cells (Figure 4C). Furthermore, mutant STAT3 cells were less sensitive to JAK inhibition (Figure 4D). In line with this observation, 6 h ruxolitinib treatment only partially reduced P-STAT3 levels in STAT3 mutant cells, but completely inhibited P-STAT3 in WT STAT3 cells (Figure 4E). Luminespib was highly effective with all cell lines (IC50<15nM) and dose-dependently reduced STAT3 Y705 phosphorylation in STAT3-mutated NK-YS cells (Figure 4F). As luminespib was highly potent in all tested Ba/F3 and NK cell lines, we wanted to assess whether the compound is generally cytotoxic. We compared the observed drug sensitivity scores (DSS) against a set of reference cell line data originating from ovarian, breast and pancreatic cancers, which were screened with the same assay at our institute. Notably, the NK and Ba/F3 cells showed almost twice as high DSS values suggesting that lymphoid cell lines that are dependent on persistent JAK/STAT signaling either induced by IL2 or STAT3 mutations are particularly sensitive to Hsp90 inhibition (Figure 4H).

Next we tested the combinatorial effect of Hsp90, mTOR or JAK inhibition against the mutant STAT3 NK-YS and YT cell lines. Highest synergy was observed between mTOR and JAK inhibitors (Supplementary Figure 5). Luminespib had a very narrow therapeutic window, and synergism was only observed at 10 nM concentration when combined with ruxolitinib or INK128 (Figure 4I, Supplementary Figure 5).

### Primary LGL leukemia cells with STAT3 mutation show increased sensitivity to Hsp90 inhibition

Up to 40% of LGL leukemia patients harbor a STAT3 mutation[9], and therefore represent a population in need of novel therapies that can block mutant STAT3 associated cell survival. To further confirm our initial findings from the cell line models on primary cells, we tested eight T-cell LGL-leukemia patient samples against mTOR, Hsp90 and JAK inhibitors. Four of the samples had heterozygous STAT3 mutations (3×Y640F, 1×D661V) in the CD8+ cell compartment with variant allele frequency of 27-51%. Detailed patient information is presented in Supplementary Table 3.

**Figure 5:**
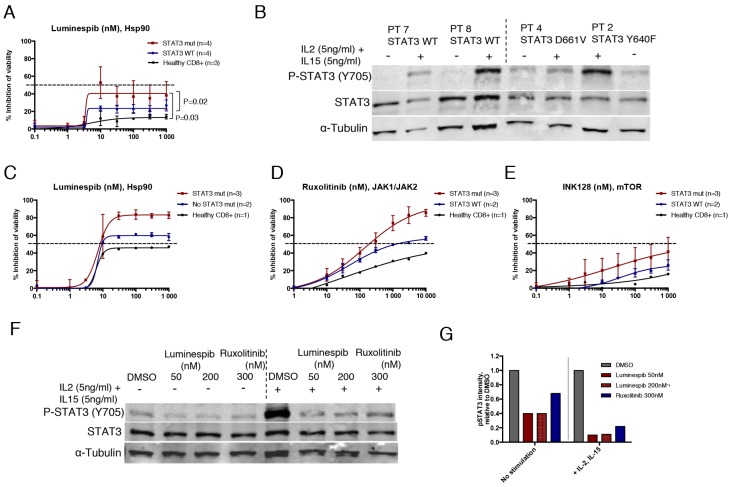
Hsp90 inhibition abrogates STAT3 phosphorylation and viability of mutant STAT3 harboring LGL leukemia patient samples **(A)** Dose response curves representing cell viability inhibition of eight T-cell LGL leukemia patient samples and three healthy controls treated with luminespib for 3 days. Cells were purified with CD8+ magnetic beads. Error bars represent the mean ±SD of patient groups with or without STAT3 mutations (n=4) or healthy controls (n=3). (*P*-values, Mann-Whitney U-test). **(B)** Western blot analysis showing Y705 P-STAT3 and total STAT3 protein levels of four LGL leukemia samples of which two harbored STAT3 mutations and two WT STAT3. Freshly purified CD8+ T-cells were incubated either in MCM media or MCM media supplemented with IL2 (5 ng/ml) and IL15 (5 ng/ml) for 1 h after protein lysates were prepared. **(C-E)** Cell viability inhibition of purified CD8+ LGL-leukemia patient cells treated 72 h with luminespib, ruxolitinib and INK128. Cells were cultured in the presence of IL2 (5 ng/ml) and IL15 (5 ng/ml). **(F)** Western blot analysis showing Y705 P-STAT3 and total STAT3 protein levels of patient 3 (Y640F, 42%) after treatment with luminespib or ruxolitinib with indicated concentrations for 16 h. Half of the cells were cultured in MCM and the other half in MCM + IL2 (5 ng/ml) and IL15 (5 ng/ml). **(G)** P-STAT3 band intensities of western blot images were quantified using Odyssey imaging system application software. P-STAT3 intensities were normalized to α-Tubulin and are relative to the DMSO control. Cells cultured with or without interleukins were normalized to their own DMSO controls.

In the absence of cytokine stimulation, results from the 3-day cell viability assay demonstrated that purified CD8+ LGL cells with mutated STAT3 were significantly more sensitive to luminespib compared to WT STAT3 LGL cells or healthy donor CD8+ cells (Figure 5A). Interestingly, ruxolitinib and INK128 were not effective suggesting that Hsp90 inhibition reduces cell viability of mutant cells even in the absence of JAK kinase activity (Supplementary Figure 6). IL2 and IL15 activate lymphoproliferative signaling through JAK1/JAK3-STAT3/STAT5 and IL15 has shown to be elevated in large granular lymphocytic leukemia [33, 34]. Western blot analysis confirmed that STAT3 was only phosphorylated in mutant STAT3 LGL cells, while addition of IL2 and IL15 stimulated STAT3 phosphorylation in all samples (Figure 5B). Cytokine stimulation increased sensitivity of the LGL cells to luminespib and conferred sensitivity to ruxolitinib and a lesser extent to INK128 (Figure 5C-5E). Notably, STAT3 mutated samples became more sensitive to the tested compounds after stimulation (Figure 5C-5E). At the protein level, luminespib and to a smaller extent ruxolitinib decreased STAT3 phosphorylation in STAT3 mutated LGL cells in the absence of cytokine stimulation, and the effect was more evident in the presence of IL2 and IL15 (Figure 5F and 5G). Taken together, our data demonstrate that Hsp90 inhibition effectively reduces the cell viability and phosphorylation of STAT3 mutated primary LGL-leukemia cells even in absence of cytokine stimulation and its effect is further increased when JAK/STAT signaling is activated with IL2 and IL15.

## DISCUSSION

Mutation to STAT3 is frequently observed in lymphoproliferative malignancies, suggesting an important role of activated STAT3 in the pathogenesis of these diseases and a potential target for therapy [8-18]. Using different cell model systems, we investigated if mutant STAT3 cells have a distinct drug sensitivity profile compared to WT STAT3 cells and searched for drugs that could effectively target STAT3 activity. Using a STAT3 luciferase reporter assay, we identified four drug classes, JAK, mTOR, CDK and Hsp90 inhibitors amongst 306 approved and investigational compounds with greatest potency against both mutant and WT STAT3 activity. We extended these results to models of STAT3-driven lymphoproliferative malignancies demonstrating that Hsp90 inhibition effectively reduced both mutant and WT STAT3 phosphorylation and viability of NK cell leukemia/lymphoma cell lines and LGL leukemia patient samples with naturally occurring STAT3 mutations.

From our functional assay, we found that JAK inhibitors were not effective at inhibiting non-stimulated mutant STAT3-driven luciferase reporter activity and STAT3 phosphorylation. In concordance, ruxolitinib treatment of heterozygous mutant STAT3-harboring NK cell lymphoma/leukemia cell lines reduced STAT3 phosphorylation to a smaller extent compared to WT cells. However, in lymphoproliferative disorders, JAK kinases activated by genetic alterations or cytokine stimulation are presumably responsible for mutant STAT3 hyperactivation [8, 34]. We found that JAK inhibitors decreased the viability and phosphorylation of STAT3-mutated NK cell lines and LGL leukemia patient samples in the presence of cytokines, indicating that STAT3 mutation alone does not confer high resistance to JAK inhibition in these conditions. In conclusion, while JAK inhibitors appear less effective in cells expressing mutant STAT3, it is likely that JAK inhibitors may nevertheless be a viable therapeutic strategy *in vivo* in STAT3-mutated malignancies through inhibition of microenvironmental cytokine stimulation and STAT3 hyperactivation. A recent clinical investigation has shown the JAK1/JAK3 inhibitor tofacitinib to be a promising salvage therapy for refractory T-LGL leukemia patients with or without STAT3 mutations [35]. However, the basal activity of mutant STAT3 can be challenging to inhibit only with JAK inhibitors and thus inhibition of mutant STAT3 through other target molecules and drug combinations could be required.

Hsp90 is a chaperone protein involved in the activation and stabilization of a wide variety of client proteins including members of the JAK/STAT and PI3K/AKT/mTOR pathways [36, 37]. We demonstrated that luminespib was able to reduce STAT3 phosphorylation and transcriptional activity of not only IL-R/JAK stimulated WT, but also non-stimulated mutant STAT3. Notably, luminespib decreased baseline Y705 phosphorylation of mutant STAT3 in the HEK293 reporter cell line in contrast to ruxolitinib. The efficacy of Hsp90 inhibition in JAK2/STAT-driven diseases such as myeloproliferative neoplasms has been previously demonstrated, but linked primarily to increased JAK2 degradation [38]. Furthermore, Hsp90 inhibitor treatment of classical Hodgkin lymphoma cells was shown to cause inhibition of STAT1, -3, -5, -6 tyrosine phosphorylation probably as a result of decreased expression and phosphorylation of JAK family members [39]. One plausible explanation is that Hsp90 inhibition is associated with the degradation of several client proteins, including JAK kinases, and thus inhibits a broad range of targets, some of which might be needed for mutant STAT3 phosphorylation and stabilization. Consistent with these observations, we showed that STAT3 mutated primary LGL-leukemia cells were sensitive to luminespib even when ruxolitinib did not show efficacy. Although, the high sensitivity of lymphoid cells to Hsp90 inhibition observed here likely depends on several factors, based on our results, Hsp90 inhibitors may be particularly effective in JAK/STAT3-driven lymphoid diseases. Altogether, Hsp90 inhibition is an effective way to inhibit persistent JAK/STAT3 signaling induced either by cytokines or STAT3 mutation, and combining Hsp90 with JAK or mTOR inhibitors can decrease STAT3 activity even further.

Other drugs classes that reduced STAT3-driven reporter signal were mTOR and CDK inhibitors. A previous study suggested that mTOR can directly phosphorylate STAT3 at the S727 residue, thus enhancing STAT3 activity [40, 41]. However, we did not observe reduced S727 or Y705 STAT3 phosphorylation suggesting that in our experiments mTOR inhibitors decreased STAT3-driven gene expression through a different mechanism such as at the transcriptional level. Our observation that mutant STAT3-dependent Ba/F3 cells and Y640F STAT3-mutated NK cell lines had increased sensitivity to rapalogs demands further investigation to understand the possible mechanistic link between STAT3 and mTOR activity.

In conclusion, our results suggest that STAT3 mutation status can affect drug sensitivity and should be considered when studying targeted therapy options in the spectrum of diseases where STAT3 mutations have been identified. We highlight Hsp90 inhibitors as particularly effective in targeting both mutant and WT STAT3-driven lymphoid malignancies and the combination of Hsp90 inhibitors with JAK or mTOR inhibitors may represent a more potent, long-term therapeutic strategy for STAT3 mutated disease.

## MATERIALS AND METHODS

### Cell lines, primary LGL cells and culture conditions

HEK293 cells containing a luciferase reporter under the control of a STAT3-responsive sis-inducible element (HEK293-SIE; Promega, Madison, WI, USA) were cultured in high-glucose DMEM containing 10% FBS, 2 mM L-glutamine, 100 U/ml penicillin and 100 μg/ml streptomycin (complete medium). Murine IL3-dependent B-cells (Ba/F3; Leibniz Institute, DSMZ, Braunschweig, Germany) were cultured in complete RPMI medium (Gibco; Thermo Scientific, Carlsbad, CA, USA) + 10 ng/ml mouse IL3 (Peprotech, Rocky Hill, NJ, USA). The Ba/F3-gp130-hIL-6R cell line was a generous gift from Christoph Garber’s laboratory (University of Kiel) and was cultured in complete RPMI + 10 ng/ml human IL6 (Peprotech). NK cell lines KHYG-1, NK-92 and YT were obtained from DSMZ. KAI3, NK-YS and SNK6 were a generous gift from Dr. John Chan (City of Hope Medical Center) and NKL from Dr. Thomas P. Loughran (University of Virginia). All NK cell lines were cultured in RPMI complete medium + 2.5 ng/ml recombinant human IL2 (BD Pharmingen, San Diego, CA, USA). Large granular lymphocytic leukemia samples were obtained from eight patients, which had had elevated levels of CD3+CD8+ cells for more than 6 months (patient information is summarized in Supplementary Table 3). The Helsinki University Hospital ethics committee approved the study, written informed consent was obtained prior to sample collection, and the samples collected according to the principles of the Declaration of Helsinki. Mononuclear cells were isolated from peripheral blood by Ficoll gradient centrifugation (GE Healthcare, Little Chalfont, Buckinghamshire, UK). CD8+ cells were subsequently enriched with CD8+ specific magnetic beads using an autoMacs instrument (Miltenyi Biotec, Bergisch Gladbach, Germany). Cells were cultured in mononuclear cell media (MCM, PromoCell, Heidelberg, Germany) supplemented with IL2 (5ng/ml) and IL15 (5ng/ml) as indicated.

### Virus production and transduction of Ba/F3 and HEK293-SIE cells

Mutant Y640F and wild type (WT) STAT3 containing plasmids were produced as previously described [9]. Amplified STAT3 was inserted into the LeGO-iCER2 plasmid (a gift from Boris Fehse, Addgene plasmid #27346) after digestion with SbfI-HF and NotI-HF restriction enzymes (New England Biolabs, Ipswich, MA, USA). Lentivirus particles were generated by co-transfecting HEK293T cells with pCMV-ΔR8.74 (5.6 μg), pCMV-VSV-G (3.6 μg) and LeGO-iCER2-STAT3 plasmid (8.6 μg) in a 10 cm dish using FugeneHD transfection reagent (Promega). The next day the medium was discarded and replaced with fresh DMEM. The supernatant was collected 2 times during 72 h, after which it was concentrated by ultracentrifugation. Ba/F3 cells were transduced with the empty plasmid (EP), human Y640F or WT STAT3 containing lentiviruses in 6-well plates, 5×10^5^ cells/well using 8 μg/ml polybrene. IL3 was removed after two days causing EP and WT STAT3 transduced cells to die. HEK293-SIE cells were similarly transduced to generate cell lines stably expressing Y640F or WT STAT3.

### Drug sensitivity assessment

A panel of 306 compounds including most approved and investigational oncology drugs as well as three small molecule STAT3 inhibitors (Stattic, LLL12, STA-21) was used for the functional assays (Supplementary Table 1). All drugs were tested in 5-8 different concentrations covering a 10 000-fold concentration range [42]. The efficacy of a drug was measured as a drug sensitivity score (DSS), which is a quantitative drug response metric based on the optimized area under the dose response curve [43]. Drug combination synergy was quantified with the synergy finder software using ZIP or HSA reference models [44].

### STAT3-mediated luciferase reporter assay

HEK293-SIE reporter cells stably expressing either WT or mutant Y640F STAT3 were dispensed to prepared 384-well drug plates at 10 000 cells/well using a MultiDrop Combi peristaltic dispenser (Thermo Fisher, Carlsbad, CA, USA). Before plating, IL6 was added directly to the cell suspension (100 ng/ml) of WT STAT3 expressing cell lines. The cells were incubated with the drugs for 6 h after which the ONE-Glo luciferase reagent (Promega) was added according to the manufacturer’s protocol. The luciferase signal was measured with a PHERAstar FS plate reader (BMG Labtech, Ortenberg, Germany).

### Cell viability assessment

Transduced Ba/F3 cells (2 000 cells/well), NK cell lines (3 000 cells/well) and primary CD8+ large granular lymphocytic leukemia cells (10 000 cells/well) were added to the prepared 384-well drug plates. After 72 h incubation at 37 °C, 5% CO_2_, viability was measured with the CellTiter-Glo (CTG) luminescent reagent (Promega) and PHERAstar FS plate reader.

### Gene silencing experiments

HEK293-SIE cells stably expressing Y640F or WT STAT3 were plated on 384-well plates in complete DMEM medium. The plates contained pre-plated siRNAs, 3x siRNA/gene in triplicate (Supplementary Table 4) and transfection reagent as follows: 250 nl of siRNAs from the Ambion® siRNA library and 5 μl of Lipofectamine RNAiMAX (Life Technologies; Thermo Fisher), pre-diluted 1:100 in OptiMEM and resulting in a final 10 nM siRNA concentration with cell density of 2 000 cells/well. The cells were incubated 72 h after which the STAT3 WT cells were induced with IL6 (100 ng/ml) and incubated for an additional 6 h. To measure cell viability CellTiter-Fluor (CTF, Promega) was dispensed on the assay plate 1.5 h prior to reading the fluorescence intensity with a PHERAstar FS plate reader. Luciferase reporter activity was measured with the ONE-Glo reagent according to the manufacturer’s instructions and read with the LUM plus filter. Luciferase activity was normalized against cell viability and the inhibitory effect of gene knockdown was measured relative to the mock siRNA experiments. Both cell lines were normalized separately to their own controls.

### Western blot analysis

Cells were lysed in RIPA buffer (Cell Signaling Technology, Danvers, MA, USA), the lysates were sonicated and the protein concentrations determined using the Qubit™ Fluorometer (Life Technologies; Thermo Fisher), after which 20-50 μg of protein was loaded per sample to 10% or 12% SDS-PAGE gels. After electrophoresis, the proteins were transferred to nitrocellulose membranes (Bio-Rad, Hercules, CA, USA), and the membranes blocked with 5% bovine serum albumin (BSA) for 1 h. Primary antibodies (anti-STAT3 cat. 2972, anti-pSTAT3 Y705 cat. 9131 anti-pSTAT3 S722 cat. 9134, anti-AKT cat. 2920, anti-AKT S473 cat. 4058 from Cell Signaling Technology; anti- β-actin cat. 20-33 from Sigma-Aldrich, St. Louis, MO, USA) were diluted 1:1 000 in Tris-buffered saline and 0.1% Tween 20 (TBS-T) + 5% BSA and incubated with the membranes 1 h at room temperature (RT) or O/N at +4°C. Secondary infrared antibodies (IRDye 800CW and IRdye680LT from LI-COR Biosciences, Lincoln, NE, USA) were diluted 1:15 000 in TBS-T + 5% BSA, and incubated with the membranes for 1 h at RT. The proteins were visualized with the Odyssey imaging system (LI-COR Biosciences) and its application software version 3.0 was used to quantify band intensity levels.

### Statistical analysis

Statistical analyses were performed with Prism software version 6.0 (GrpahPad Software, San Diego, CA, USA). Differences between responses were calculated by using the Mann-Whitney *U* test. *P*-values of <0.05 were considered as statistically significant.

## SUPPLEMENTARY MATERIALS FIGURES AND TABLES








