# Supplementary material for: Drug sensitivity profiling identifies potential therapies for lymphoproliferative disorders with overactive JAK/STAT3 signaling
Source: Oncotarget. 2017 Oct 31;8(57):97516–27. doi: 10.18632/oncotarget.22178 (PMC5722580; doi:10.18632/oncotarget.22178)
Supplement: Supplementary file 1 [file oncotarget-08-97516-s001.pdf]

Drug sensitivity profiling identifies potential therapies for lymphoproliferative disorders with overactive JAK/STAT3 signaling

SUPPLEMENTARY MATERIALS

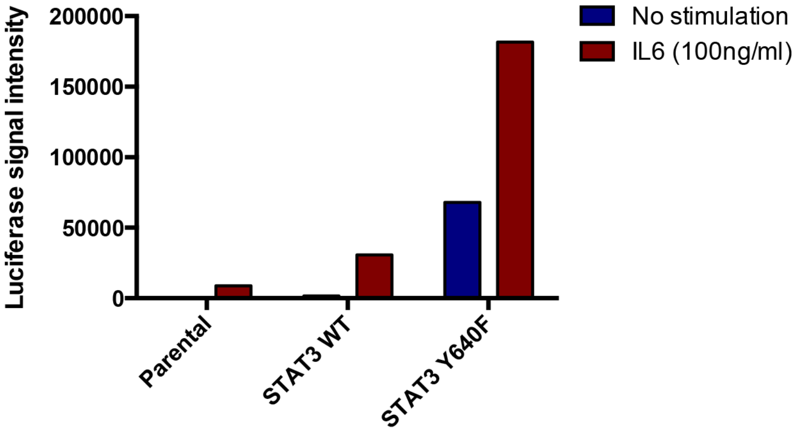

Supplementary Figure 1: Basal and IL-6 induced luciferase activity of different HEK-SIE cell line models. Parental HEK-SIE cells were transduced either with WT or Y640F mutant STAT3. Part of the cells was induced with IL6 (100ng/ml) for 6 h after which the luciferase signal intensity was measured. Results indicate that in the absence of IL6 stimulation of WT STAT3, basal luciferase activity is minimal, whereas mutant STAT3 is constitutively active and is hyperactivated upon IL6 stimulation.

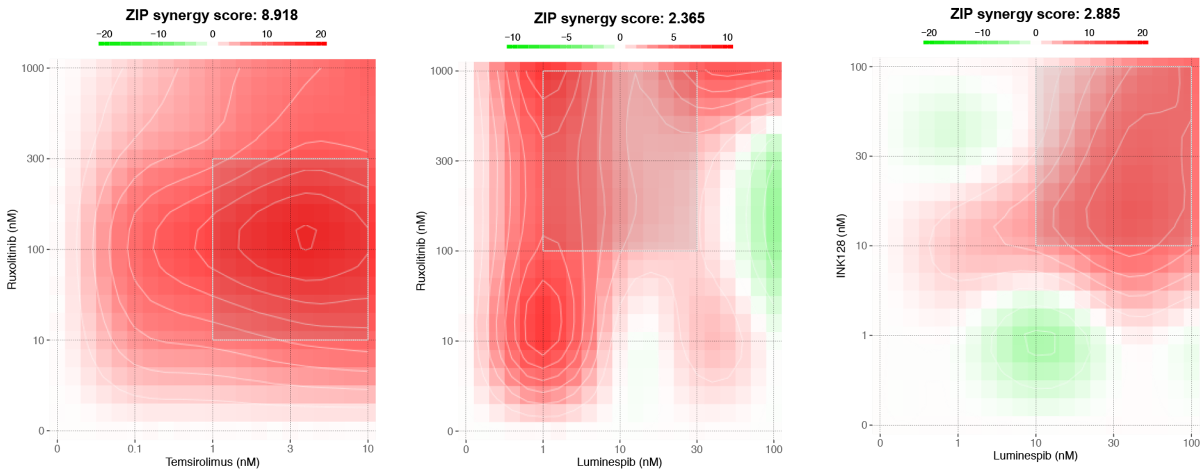

Drug Combination	Most synergistic area score (ZIP)	Most additive area score (HSA)
Temsirolimus-Ruxolitinib	11.75	16.49
INK128-Ruxolitinib	0.09	8.07
Luminespib-Ruxolitinib	4.26	17.23
Luminespib-INK128	6.36	14.53
Luminespib-Temsirolimus	-1.71	-1.02

Supplementary Figure 2: Dual combinations with luminespib and INK128 or ruxolitinib synergistically inhibit mutant STAT3 activity. HEK293-SIE reporter cells expressing mutant STAT3 were treated with increasing concentrations of the two compounds, incubated for 6 hours, and then the luciferase signal measured. The results are presented in 2-D plots with red color indicating drug synergism and green color antagonism. Synergism was calculated using the ZIP method and the additive score was calculated using the HSA method. The additive/synergistic area scores presented in the table were calculated from concentrations within the overlying squares in the 2-D plots.

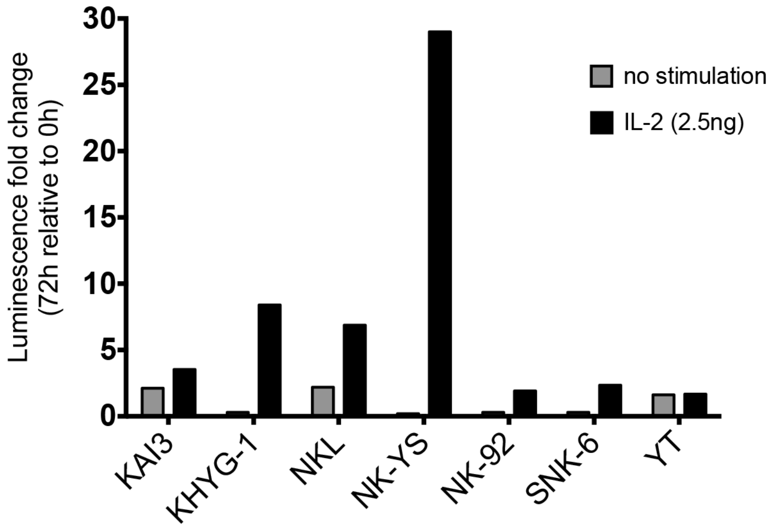

Supplementary Figure 3: Proliferation and survival of NK-cell lines are dependent on IL2. Cell lines were treated with or without IL2 (2.5ng/ml). Viability was measured at day 0 and day 3 with CTG cell viability assay. Fold change indicates the day 3 luminescence signal relative to day 0.

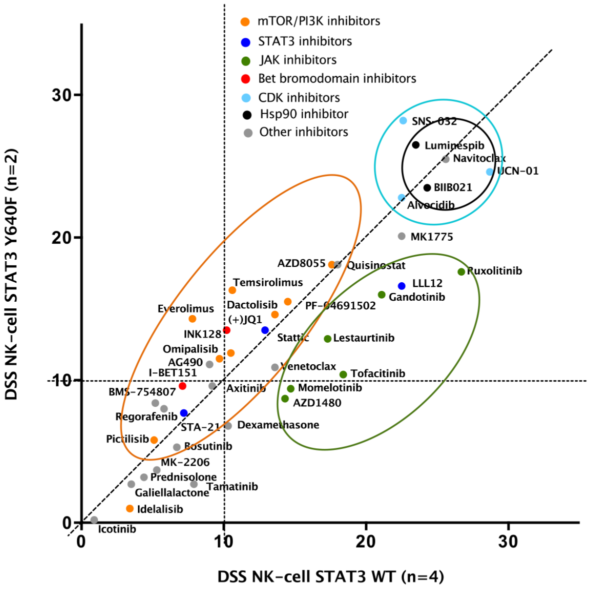

Supplementary Figure 4: Mutant STAT3 NK cells show increased mTOR and decreased JAK inhibitor sensitivity. Results are shown as a drug sensitivity score (DSS), which were calculated from the dose response curves generated from the viability of the NK cell lines measured after 3-day incubation with the compounds using the CTG assay. Y-axis: mean DSS of NK cell lines harboring the Y640F STAT3 mutation (YT, NK-YS). X-axis: mean DSS of NK cell lines with WT STAT3 (KAI3, NK-92, NKL, KHYG-1). The experiment was repeated twice.

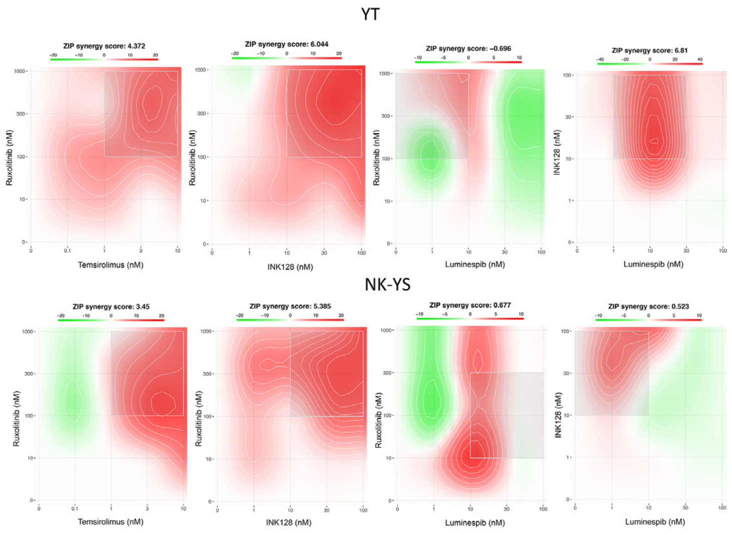

Drug Combination	Most synergistic area score (ZIP) NK-YS	YT
Temsirolimus-Ruxolitinib	7.2	6.5
INK128-Ruxolitinib	8.0	9.8
Luminespib-Ruxolitinib	2.0	*0.8*
Luminespib-INK128	1.3	*11.1*
Luminespib-Temsirolimus	0.3	3.9

Supplementary Figure 5: Dual combinations with luminespib, INK128, ruxolitinib and temsirolimus synergistically inhibit the viability of NK cell lines with mutant STAT3. The NK-YS and YT cell lines were treated with increasing concentrations of the indicated two compounds for 3 days and viability measured with the CTG assay. The results are presented in 2-D plots with red color indicating synergism and green antagonism. Synergism was calculated using the ZIP method and the additive score was calculated using the HSA method. The additive area/synergism scores presented in the table were calculated from the concentrations within the overlying squares in the 2-D plots.

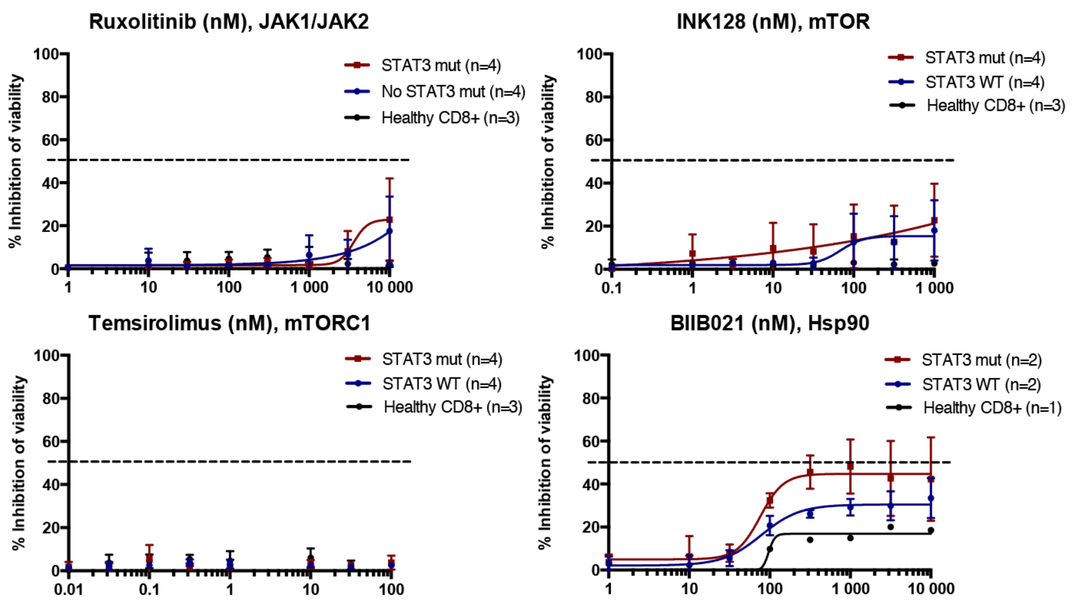

Supplementary Figure 6: Dose response curves representing cell viability inhibition of eight LGL-leukemia patient samples and three healthy controls treated with ruxolitinib, INK128, temsirolimus or BIIB021 for 3 days. CD8+ cells were purified by immunomagnetic bead separation and cultured in MCM media. Error bars represent the mean ±SD of the patient groups with or without STAT3 mutations or healthy donors.

Supplementary Table 1: (A,B) Drug and compound collection used in the drug sensitvity and resistance testing assay.

See Supplementary File 1

Supplementary Table 2: (A) Dose reponse curves, DSS and IC50 values for the tested 62 drugs in the HEKSIE cell lines (6 h treatment). (B) Dose reponse curves, DSS and IC50 values for the tested 62 drug on NK lymphoma cell lines.

See Supplementary File 2

Supplementary Table 3: The main characteristics of LGL leukemia patients.

See Supplementary File 3

Supplementary Table 4: siRNA IDs and sequences

RefSeq Accession Number	Gene Symbol	Gene ID	siRNA ID	Sense siRNA Sequence
**NM_004958**	FRAP1	2475	s604	GGAGCCUUGUUGAUCCUUAtt
**NM_004958**	FRAP1	2475	s602	GCUCGUAGUUGGGAUAACAtt
**NM_004958**	FRAP1	2475	s603	CAUUCGCAUUCAGUCCAUAtt
**NM_002227**	JAK1	3716	s7647	GUAUUAAGCUCAUCAUGGAtt
**NM_002227**	JAK1	3716	s7648	GGUGCUGUCUAGGCAAGAAtt
**NM_002227**	JAK1	3716	s7646	CCAUCACCGUUGAUGACAAtt
**NM_004972**	JAK2	3717	s7650	GGACUGUAUGUACUUCGAUtt
**NM_004972**	JAK2	3717	s7651	CCAGCGGAAUUUAUGCGUAtt
**NM_004972**	JAK2	3717	s7649	CAAAGAUCCAAGACUAUCAtt
**NM_000215**	JAK3	3718	s7653	GUAUCGUGGUGUCAGCUAUtt
**NM_000215**	JAK3	3718	s7654	GGAGAUUCAGAUCCUCAAAtt
**NM_000215**	JAK3	3718	s7652	CUAAGAAACUCCAAUUUUAtt
**NM_003150**	STAT3	6774	s745	GCACCUUCCUGCUAAGAUUtt
**NM_003150**	STAT3	6774	s744	GGCUGGACAAUAUCAUUGAtt
**NM_003150**	STAT3	6774	s743	GCCUCAAGAUUGACCUAGAtt
**NM_003299**	HSP90B1	7184	s14473	GUACAUCCCUCUGUCCAUAtt
**NM_003299**	HSP90B1	7184	s14474	GCAUUGGACUUGCUUGGAAtt
**NM_003299**	HSP90B1	7184	s14475	CCAUCGGUGCAAAUUUACAtt
**NM_003299**	HSP90AA1	3320	s6993	UGAGAUUUGUCGAACCAAAtt
**NM_003299**	HSP90AA1	3320	s6994	CCGCUGCAAGGGUAGUAUAtt
**NM_003299**	HSP90AA1	3320	s6995	GAAGUUUCCAAAUACGAGAtt
